# Arsenic in Eggs and Excreta of Laying Hens in Bangladesh: A Preliminary Study

**DOI:** 10.3329/jhpn.v30i4.13290

**Published:** 2012-12

**Authors:** Amalendu Ghosh, M. A. Awal, Shankar Majumder, Mahbub Mostofa, Abul Khair, M. Z. Islam, D. Ramkishan Rao

**Affiliations:** ^1^Upazila Livestock Office, Netrokona Sadar, Netrokona, Bangladesh; ^2^Department of Pharmacology, Bangladesh Agricultural University, Mymensingh, Bangladesh; ^3^Department of Agricultural Statistics, Bangladesh Agricultural University, Mymensingh, Bangladesh; ^4^Department of Pharmacology, Bangladesh Agricultural University, Mymensingh, Bangladesh; ^5^District Livestock Office, Noakhali, Bangladesh; ^6^Department of Pharmacology, Bangladesh Agricultural University, Mymensingh, Bangladesh; ^7^National Institute of Food and Agriculture-USDA, USA

**Keywords:** Arsenic, Drinking-water, Egg, Excreta, Feed, Laying hen, Bangladesh

## Abstract

The aim of this study was to detect arsenic concentrations in feed, well-water for drinking, eggs, and excreta of laying hens in arsenic-prone areas of Bangladesh and to assess the effect of arsenic-containing feed and well-water on the accumulation of arsenic in eggs and excreta of the same subject. One egg from each laying hen (n=248) and its excreta, feed, and well-water for drinking were collected. Total arsenic concentrations were determined by atomic absorption spectrophotometer, coupled with hydride generator. Effects of arsenic-containing feed and drinking-water on the accumulation of arsenic in eggs and excreta were analyzed by multivariate regression model, using Stata software. Mean arsenic concentrations in drinking-water, feed (dry weight [DW]), egg (wet weight [WW]), and excreta (DW) of hens were 77.3, 176.6, 19.2, and 1,439.9 ppb respectively. Significant (p<0.01) positive correlations were found between the arsenic contents in eggs and drinking-water (r=0.602), drinking-water and excreta (r=0.716), feed and excreta (r=0.402) as well as between the arsenic content in eggs and the age of the layer (r=0.243). On an average, 55% and 82% of the total variation in arsenic contents of eggs and excreta respectively could be attributed to the variation in the geographic area, age, feed type, and arsenic contents of drinking-water and feed. For each week's increase in age of hens, arsenic content in eggs increased by 0.94%. For every 1% elevation of arsenic in drinking-water, arsenic in eggs and excreta increased by 0.41% and 0.44% respectively whereas for a 1% rise of arsenic in feed, arsenic in eggs and excreta increased by 0.40% and 0.52% respectively. These results provide evidence that, although high arsenic level prevails in well-water for drinking in Bangladesh, the arsenic shows low biological transmission capability from body to eggs and, thus, the value was below the maximum tolerable limit for humans. However, arsenic in drinking-water and/or feed makes a significant contribution to the arsenic accumulations in eggs and excreta of laying hens.

## INTRODUCTION

Arsenic toxicity is a global health problem affecting millions of people. It is present ubiquitously in the environment and is released from both natural and man-made sources (1). Drinking-water is now recognized as the major source of human intake of arsenic in its most toxic (inorganic) forms. The arsenic disaster of Bangladesh has been called the most terrible environmental catastrophe of the twentieth century. The extent of this environmental disaster is greater than any other recorded in human history (2). It was estimated that groundwater in 59 out of 64 districts of Bangladesh is contaminated with arsenic (3).

Several studies indicated that irrigation with contaminated groundwater have significantly increased arsenic levels in agricultural soils (4,5). Food crops grown on arsenic-contaminated soil can accumulate high levels of arsenic in seeds, stem, and leaves, which adds arsenic in food-chain of man and animals (4,6). Bangladesh populations are getting arsenic mainly through drinking contaminated tubewell-water and through consumption of contaminated foods. Chronic intake of arsenic is strongly associated with an increased risk of liver, kidney, skin and lung cancer (7,8); cardiovascular disease (9,10); and an increase in the mortality rate (11). Besides these, many other toxic effects of arsenic have been reported. Examples are its compromising effect on some immune functions (12-14), inhibition of neurite growth (15), and oxidative myocardial and liver injury (16,17). WHO set the permissible limit of arsenic in drinking-water at 10 ppb (18). For Bangladesh, this is 50 ppb.

Poultry provides hard-cash income and creates employment opportunities for the rural and landless farmers in Bangladesh. Layer hens are now providing an unprecedented range of relatively low-cost egg and meat products for consumers. At present, most poultry farms in Bangladesh are being maintained with shallow well-water which contains relatively more arsenic than deep well-water (3). Hens raised with arsenic-rich drinking-water and feed may accumulate arsenic residue in their flesh, eggs, and excreta, which potentially leads to arsenic in the human food-chain (19). Public-health workers have expressed concern about the arsenic content of chicken meat for its role as human diet (20). Moreover, arsenic has been identified as a roadblock to potential solutions in animal waste management (21). The bioavailability of arsenic in foods, especially meat and eggs, and the arsenic risks to human health need to be assessed carefully. No data exist in Bangladesh on the level of arsenic in eggs and excreta of hens fed arsenic-rich feed and/or drinking-water. Thus, the objectives of the present study were to determine the arsenic concentration in well-water for drinking, feed, eggs, and excreta of laying hens in arsenic-prone areas of Bangladesh, and to assess the effect of arsenic in drinking-water and feed on the accumulation of arsenic in eggs and excreta of laying hens.

## MATERIALS AND METHODS

### Study design

A restricted observational (survey) study design was used in this work.

### Sampling site

For this study, five worse arsenic-affected districts (Madaripur, Chandpur, Jessore, Satkhira, and Faridpur) of Bangladesh were selected, and field samples were collected from these districts. Information about these severely arsenic-contaminated areas was obtained from secondary sources (22) where concentrations of arsenic exceeding 1,000 ppb were reported in a few shallow tubewells.

### Sample-collection procedure

A multistage sampling procedure was used for selecting the ultimate sample of the laying hens. In total, 62 layer farms were selected purposively from the aforesaid areas on the basis of pre-defined criteria of age (20-80 weeks) and availability of records on the source of feed and drinking-water; then, 248 commercial laying hens—four from each farm—were selected randomly. Samples were collected during March-May 2009 by interviewing the farm-owners based on a pre-arranged questionnaire. The questionnaire was pre-tested and finalized after incorporation of feedback. Information about area, age, and history of feed and water consumption by the hens, including water source and depth of tubewells, were obtained on the basis of the questionnaire.

### Drinking-water for hens

Tubewell-water samples consumed by hens were collected in 50 mL acid-washed polyethylene vial as previously described (23). Briefly, water samples were collected from mid-stream after pumping the tubewells vigorously for at least 10 minutes. Immediately after collection, samples were filtered with capsule filter (0.45 µm pore); then, the collecting vials were flushed with filtered water for about one minute. Vials were filled up to the top by filtered water. An aliquot of 100 µL concentrated nitric acid was added (at the field prior to water collection) to acidify the sample to a pH <2 and to prevent precipitation of iron and co-precipitation of arsenic (24).

### Poultry feed

About 50 g feed (commercially-prepared or home-made with local ingredients), used as ration for chickens, was collected from each selected farm for arsenic analysis.

### Eggs and excreta

With the consent of the proprietor, four laying hens from each of the selected farms were kept separately, one in each cage, with the bedding of chemically-clean and dry polyethylene mat. Generally, birds were confined in cages from 8-9 am until laying and dropping. Hens were allowed free access to sampled feed and water in cages. Eggs were collected, washed in tap-water and finally rinsed with distilled water. About 10-15 g excreta from each hen was collected in sterile zipper bags. Eggs and excreta were stored (eggs at 4 ºC and excreta at −20 ºC) until analysis. When one or more layer hen(s) failed laying, egg(s) and/or excreta were collected on the subsequent day, following the procedure described, with equal numbers of birds from the respective farms.

### Sample preparation

Poultry feed were oven-dried at 60 ºC for 72 h and ground with a stainless steel grinder (Karl Kolb, Scientific Technical Supplies, West Germany). It was passed through 100-mesh sieve (pore-size 0.149 mm) and stored in desiccators. Before digestion, this was further oven-dried at 50 ºC to get constant weight. About 0.3-0.5 g (DW) sample was taken into digestion tube. This was digested by block digester (M-24 plazas/samples, JP Selecta, Spain), using concentrated HNO_3_ and 30% H_2_O_2_, heating at 120 ºC (25). For egg sample preparation, each egg was broken separately; yolk and albumin were homogenized by mixing in a blender. An aliquot of 3-4 mL (WW) egg homogenate was transferred to digestion tube. Samples were acid-digested in block digester, heating up to 150 ºC by sequentially adding 5-7.5 mL triple acid mixture (HNO_3_-10 parts, HClO_4_-3 parts, and H_2_SO_4_-1 part) and 3 mL H_2_O_2_ in each digestion tube (26). Excreta samples were dried in oven at 50 ºC and ground for homogeneity by a blender. To remove coarse particles, blended samples were passed through one-mm sieve and stored in desiccators. Before analysis, it was further dried at 60 ºC in oven until reaching a constant weight. About 0.2-0.3 g (DW) ground excreta sample was taken in each digestion tube. Samples were digested using tri-acid mixture and H_2_O_2_ as egg homogenate (26). For complete digestion of carbonaceous material, heating was continued until colourless clear watery solution appeared. Finally, the digested samples were diluted to 50 mL by adding Millipore water and filtered through filter paper (Whatman No. 41). To validate the assay, every fifteenth sample—one blank and one standard reference material (SRM)—were digested as sample, following the same digestion procedures.

### Arsenic analysis

Arsenic pentoxide (As_2_O_5_ (1,000,000,1,000) ppb; Merck, Germany) was used as standard. It was reduced to trivalent state for constructing standard curve (27). One percent HCl was used as carrier liquid. Concentrations of arsenic in drinking-water and digested samples were determined using atomic absorption spectrophotometer, coupled with hydride generator (PG Instruments Ltd., UK), following pre reduction with potassium iodide (KI) and potassium borohydride (KBH_4_) to generate arsine (AsH_3_) (28). Detection limit of the instrument for arsenic was two ppb. Quantification of arsenic was performed by spiking samples with working standard of 0, 2.5, 5, 10, 15, and 20 ppb prepared immediately before use by serial dilution of the stock in 10% HCl. Samples exceeding the standard curve range were diluted with 1% HCl to get standard curve range. The concentrations of arsenic in those samples were resolute multiplying by the dilution factor as appropriate. In every occasion, the linear correlation factor was bigger than 0.99. Determinations were performed in duplicate, having the relative error <1%. The salient features of instrument setting and carriers are summarized in Table 1.

**Table 1.** Instrumental and chemical conditions employed for the determination of arsenic by flow injection-hydride generator-atomic absorption spectrophotometer

Parameter	Instrumental and chemical condition
Light source	Ordinary hollow-cathode lamp
Measurement mode	Peak height
Carrier gas	Pure argon
Lamp current	10 mA
Wavelength	193.7 nm
Spectral bandwidth	0.4 nm
Integration time	15 sec
Delay time	5 sec
Carrier gas-flow rate	150 mL/minute
Carrier liquid	1% HCl
Blank solution	10% HCl
Reductant	1.5% KBH_4_ in 0.3% NaOH

### Quality control

Accuracy and precision of analyses were evaluated using commercially-available reference materials, with certified or recommended arsenic concentrations (Table 2). For eggs, known concentration of arsenic standard was analyzed to validate the assay. There was a good conformity between obtained arsenic concentrations in the reference materials and the reference values, signifying good analytical performance.

**Table 2.** Reference materials analyzed for arsenic, together with collected samples in the present work

Reference material (Certified or recommended values)	Matrix	Recovery rate
NIST 1643e (60.45±0.72 ppb)	Water	93 to 102%
NIST 1568a (290±30 ppb)	Poultry feed/Excreta	91 to 99%
Arsenic standard (20 ppb)	Egg	89 to 97%

### Statistical analyses

Information on laying hens was stratified according to area, age, feeding status, drinking-water source, and arsenic levels in drinking-water and feed. Multiple comparisons of means were performed using *F*-test with a significance level at p<0.05. Tukey's test was performed for multiple comparisons among the means in a particular item (29). Pearson's correlation coefficient was computed to evaluate the degree of linear relationship between two variables. Multivariate regression analysis, a logical extension of the multiple regression concept to allow for multiple responses, was done to explore the effects of the explanatory variables on dependent variables (30). Area (ref: Faridpur), age, feed type (ref: commercial feed), and arsenic contents in drinking-water and feed were considered explanatory variables, and arsenic excreted through eggs and excreta were considered dependent variables. For the multivariate analysis, arsenic contents in drinking-water, feed, eggs, and excreta were log-transformed to remove the bad impact of the extreme values. All data were analyzed using the Statistical Package for Social Sciences (SPSS, Chicago, IL). Multivariate regression analyses were carried out using Stata (version 10) software (31).

## RESULTS

Table 3 presents classification of laying hens based on the geographic area, age, sources of feed and drinking-water, and arsenic exposure levels from water and feed. Among the hens, 48.4% were fed home-made feed consisting of local ingredients while the remaining 51.6% consumed commercial feed. About two-thirds of the hens consumed shallow tubewell-water, and the remaining consumed deep tubewell-water. Nearly half of the hens (48.4%) consumed water containing up to 50 ppb of arsenic, followed by 27.4% of the hens consuming water tainted by arsenic between 51 and 100 ppb. The reminder of the hens consumed water tainted with arsenic at levels (ppb) of 101-150 (8.1%), 151-200 (4.8%), and >200 (11.3%). About 38.7%, 27.4%, and 33.9% of the hens consumed feed contaminated with arsenic levels of 100, 101-200, and >200 ppb respectively.

Arsenic content in well-water for drinking and feed supplied to the laying hens are documented in Table 4. The overall mean arsenic concentration in drinking-water was 77.3 ppb (95% CI: 66.6-88.0). Arsenic content in well-water up to 90 metre and above 90 metre depth differed significantly (p<0.01) whereas no significant difference (p>0.05) was observed between 45 metre and >45-90 metre depth. Significant difference (p<0.01) in arsenic concentration between commercially-prepared feed and the home-made feed prepared by local ingredients was also observed.

Area-wise arsenic concentrations in well-water for drinking, feed, eggs, and excreta of laying hens are summarized in Table 5. The overall mean arsenic concentration in feed (DW), eggs (WW), and excreta (DW) of layers were 176.6 ppb (95% CI: 160.9-192.3), 19.2 ppb (95% CI: 16.9-21.5) and 1,439.9 ppb (95% CI: 1,321.2-1,558.7) respectively. Arsenic concentrations in drinking-water, feed, and excreta of laying hens varied insignificantly (p>0.05) at different locations. On the other hand, arsenic content in eggs differed significantly (p<0.01) from area to area.

**Fig. 1.**
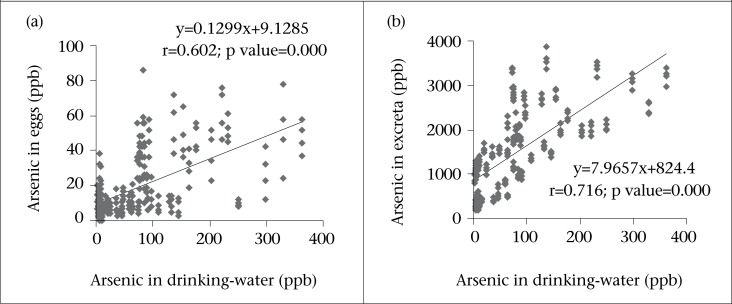
Relationships between arsenic concentrations in drinking-water and (a) eggs and (b) excreta of laying hens

**Fig. 2.**
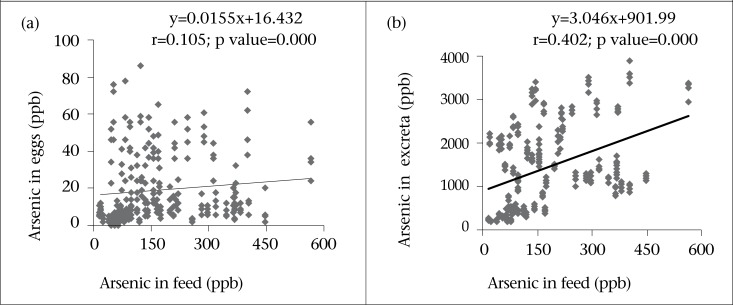
Relationships between arsenic concentrations in feed and (a) eggs and (b) excreta of laying hens

**Fig. 3.**
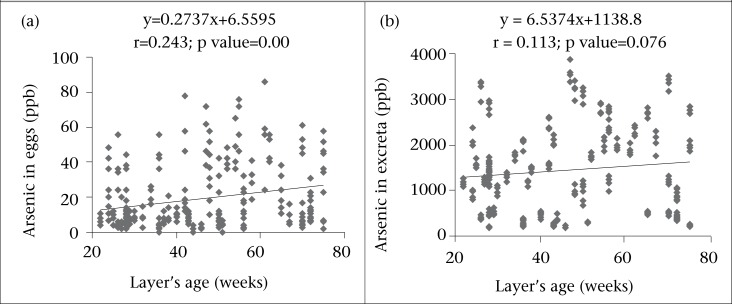
Relationships between age and arsenic concentrations in (a) eggs and (b) excreta of laying hens

Correlation between arsenic concentrations in eggs as well as excreta and drinking-water are presented in Fig. 1. The results show a strong significant (p<0.01) positive correlation between arsenic content in eggs and that in drinking-water (r=0.602) as well as excreta and drinking-water (r=0.716). Likewise, quantity of arsenic in excreta was significantly (r=0.402, p<0.01) correlated with the arsenic concentration in feed for the layers (Fig. 2). Results signify that more the arsenic in drinking-water, the more was the elevation of arsenic in eggs and excreta. Furthermore, arsenic contents in eggs was significantly (p<0.01) correlated (r=0.243) with the age of layers but an insignificant (p>0.05) correlation was observed in the arsenic content in excreta with the age of laying hens (Fig. 3).

**Table 3.** Numbers of laying hens by geographic area, age, consumption of feed type, drinking-water source, and ranges of arsenic levels in drinking-water and feed (n=248)

Characteristics	Number of subjects
Area	
Madaripur	60
Chandpur	52
Satkhira	44
Jessore	48
Faridpur	44
Age (weeks)	
Up to 30	68
>30-50	88
>50-70	64
>70	28
Feed type	
Commercially-prepared	128
Home-made by local ingredients	120
Drinking-water source	
Shallow tubewell	160
Deep tubewell	88
Arsenic level in drinking-water (ppb)	
Up to 50	120
>50-100	68
>100-150	20
>150-200	12
>200	28
Arsenic level in feed (ppb)	
Up to 100	96
>100-200	68
>200	84

The results of multivariate regression analysis are summarized in Table 6. The significant F values (and, therefore, R^2^ values) of the model reveal the perfectness of fitting the models to the data. The R^2^ values (0.55 and 0.82) imply that 55% and 82% of the total variation in arsenic content in eggs and excreta respectively could be due to the discrepancy in the area, age, feed type, and arsenic contents in drinking-water and feed. For this analysis, Faridpur was considered the reference area. Arsenic in eggs was significantly (p<0.01) higher at Madaripur compared to Faridpur. It could be expected that geometric mean of arsenic in eggs was [exp (0.45853)-1]x100=58.17% higher at Madaripur compared to Faridpur. Conversely, significantly lower (p<0.05) arsenic in eggs was observed at Chandpur compared to Faridpur, i.e. geometric mean of arsenic in eggs was 25.53% lower in Chandpur compared to Faridpur, which was [exp (-0.2947185)–1]x100=-25.53%. Arsenic contents in eggs at other localities (Satkhira and Jessore) differed insignificantly (p>0.05) with that of Faridpur. However, area exerted no significant effect on the arsenic in excreta of laying hens. A significant (p<0.01) positive impact of age on the arsenic accumulation in eggs was observed. With the advancement of one week in age, arsenic content in eggs increased, on an average, by [exp (0.0093909)-1]x100=0.94%, keeping other factors constant. However, arsenic content in excreta was not affected by age. A significant (p<0.01) monotonous increase in arsenic contents in eggs and excreta was observed with increasing magnitude of arsenic in drinking-water and feed. On average, 0.41% and 0.44% arsenic were amplified in eggs and excreta respectively for 1% elevation of arsenic in drinking-water, keeping all other factors constant. Conversely, 1% increase in the arsenic level in feed resulted in 0.40% and 0.52% elevation of arsenic in eggs and excreta respectively, keeping all other arsenic exposure items constant.

**Table 4.** Mean arsenic concentration (ppb) in tubewell-water and feed supplied to laying hens

Variable	Arsenic concentration^†^	95% CI for mean	F value
Tubewell-water			
Depth-wise (metre)			
Up to 45	114.0^a^	99.8-128.3	75.4^**^
>45-90	131.0^a^	97.0-165.0
>90	6.6^b^	6.1-7.1
Source-wise			
Shallow tubewell (≤75 metre)	116.2^a^	103.0-129.3	
Deep tubewell (>75 metre)	6.6^b^	6.1-7.1	149.6^**^
Overall	77.3	66.6-88.0	
Feed			
Commercially-prepared	88.4^b^	81.1-95.7	
Home-made by local ingredients	270.7^a^	249.8-291.7	278.8^**^
Overall	176.6	160.9-192.3	

^**^p<0.01

^†^Any two means having different superscripts differ significantly (p<0.05)

**Table 5.** Area-wise arsenic concentration (mean in ppb) in drinking-water, feed (DW), eggs (WW), and excreta (DW) of laying hens

Item	Area	Arsenic concentration^†^	95% CI for mean	F value
Drinking-water	Madaripur	90.5	64.9-116.1	
	Chandpur	63.4	40.3-86.4	
	Satkhira	57.4	40.3-74.5	1.6
	Jessore	84.7	63.1-106.2	
	Faridpur	87.6	57.1-118.0	
	Overall	77.3	66.6-88.0	
Feed	Madaripur	175.8	132.7-218.9	
	Chandpur	161.9	133.8-190.1	
	Satkhira	196.0	159.1-232.9	1.1
	Jessore	156.5	127.4-185.6	
	Faridpur	197.6	163.5-231.8	
	Overall	176.6	160.9-192.3	
Eggs	Madaripur	28.0^a^	22.1-34.0	
	Chandpur	12.2^b^	8.5-15.8	
	Satkhira	12.8^b^	9.5-16.1	7.6^**^
	Jessore	19.2^ab^	13.7-24.6	
	Faridpur	21.8^ab^	16.6-27.0	
	Overall	19.2	16.9-21.5	
Excreta	Madaripur	1,583.1	1,289.7-1,876.5	
	Chandpur	1,307.9	1,024.8-1,590.9	
	Satkhira	1,377.7	1,151.8-1,603.6	0.7
	Jessore	1,422.9	1,174.9-1,670.8	
	Faridpur	1,481.7	1,213.1-1,750.4	
	Overall	1,439.9	1,321.2-1,558.7	

^**^p<0.01

^†^Any two means having different superscripts differ significantly (p<0.05)

**Table 6.** Multivariate regression model for identifying the significant effects of arsenic exposure indices on arsenic concentrations in eggs and excreta of laying hens^†^

Regression indices and explanatory variable	Dependent variable^‡^
	Eggs	Excreta
Intercept	-1.33^**^ (0.45)	2.97^**^ (0.23)
Regression coefficients		
Area (Reference: Faridpur)		
Madaripur	0.46^**^ (0.15)	0.09 (0.08)
Chandpur	-0.29^*^ (0.15)	0.01 (0.08)
Satkhira	-0.28 (0.15)	0.02 (0.08)
Jessore	-0.22 (0.15)	0.03 (0.08)
Age (weeks)	0.01^**^ (0.00)	-0.00 (0.00)
Feed type (Reference: Commercial feed)	0.00 (0.13)	0.06 (0.07)
Arsenic in drinking-water (ppb)	0.41^**^ (0.03)	0.44^**^ (0.02)
Arsenic in feed (ppb)	0.40^**^ (0.09)	0.52^**^ (0.04)
R^2^	0.55	0.82
F value	36.83^**^	133.42^**^

^*^p<0.05

^**^p<0.01

^†^Data on arsenic concentrations in drinking-water, feed, eggs and excreta were fitted to the model after log transformation

^‡^Values in the parentheses stand for standard error

## DISCUSSION

The results of this study showed that wide ranges of arsenic are present in drinking-water, feed, eggs, and excreta of laying hens at different locations of Bangladesh. Arsenic contents in drinking-water detected in our study were higher than allowable limit of Bangladesh standard (50 ppb) but arsenic contents in eggs were within admissible limit of 500 ppb for human consumption (32). The multivariate regression models fitted to the arsenic exposure data indicated that arsenic contents in eggs and excreta of laying hens were influenced by the arsenic-contaminated feed and drinking-water.

Drinking-water was analyzed for detection of possible sources of arsenic contamination in laying hens. We found wide ranges of arsenic contamination in drinking-water, which is consistent with the previous reports on different arsenic-affected areas of Bangladesh (11,33). Arsenic concentrations in groundwater are increasing day by day throughout the world (34) and, with the progression of time, uncontaminated wells and unaffected areas are being affected, which poses a greater risk of arsenic toxicity. Increasing extraction of groundwater in Bangladesh might be a concern. Earlier reports suggest that concentration of arsenic decreases with the increment of tubewell depth (3,35). However, we got a little bit higher arsenic in tubewell-water in between >45-90 metre depth than up to 45 metre depth, although the difference was insignificant. Comparatively lower arsenic in drinking-water in our study than reported previously (11,33) and a little bit higher arsenic in well-water from >45-90 metre depth than up to 45 metre depth may be due to differences among geographical areas and small number of samples.

Detected arsenic concentration in feed for layers (176.6 ppb) was very little compared to the maximum acceptable concentration of 2,000 ppb in complete feedstuffs (36). Presently, poultry feed chiefly contains mixtures of plant-based products, like maize and soybean cake. These are mostly imported from foreign countries where arsenic in groundwater and soil may be comparatively lower than in Bangladesh. Moreover, arsenic uptake by maize from soil is very low (37), resulting in little arsenic content in most of the poultry feeds. In this study, some of the feeds used for layer hens were prepared using local ingredients of Bangladesh, like rice-bran, wheat, and fish meal as protein and energy sources. These ingredients may restrain relatively more arsenic than imported items that may substantiate the arsenic loads of feed for layers.

Relatively small amount of arsenic was detected in eggs (19.2 ppb), which were close to that reported for White Leghorn layers (24.6±0.3 to 26.4±1.2 ppb) at Taiwan (38). Presence of small amount of arsenic in eggs of the layer hens even maintained with arsenic-rich drinking-water and feed indicates its lower transmission from blood plasma to the eggs. Epidemiological and experiment data demonstrated that arsenic can readily cross the placental barrier and that significant foetal exposure may occur when mothers are exposed to arsenic (39,40). In spite of that, existence of diminutive arsenic content in eggs than FDA admissible limit of 500 ppb (32) is encouraging where arsenic catastrophe is a great problem.

Arsenic detected in excreta of laying hens in our study (1,439.9 ppb) was slightly higher than previous report on excreta (879±45 to 926±56 ppb) of 32-week old White Leghorn layers (38). However, in 1 to 7-week old broilers, 210±20 to 580±110 ppb arsenic was reported in excreta (41). Presence of relatively higher amount of arsenic in excreta in this investigation than the amounts reported previously (38,41) is possibly due to simultaneous exposure to arsenic through feed and/or drinking-water. It has been reported that the inclusion of arsenic-rich feed and drinking-water resulted in high arsenic in excreta (42). Arsenic in drinking-water is chiefly inorganic in nature (43), which is generally much more toxic than organic forms. Staple foods accumulate mostly inorganic arsenic (44) whereas sea-foods, like fish meal, contain mostly organic arsenic. Inorganic arsenic is well-absorbed in chick intestine (45) but it is excreted effectively. Organic arsenic in the form of arsenobetaine does not undergo biotransformation to other forms in animal body, and it is readily excreted through urine. Both inorganic and organic forms of arsenic substantiate the arsenic load in excreta. The use of arsenic-rich excreta and/or litter as fertilizer in agricultural fields contaminates the land (46) by elevating soil arsenic load. Long-term accumulation of heavy metals in agricultural soil has the potential to reduce soil productivity by inhibiting soil microbial populations (47) that may pose a risk to the ecosystem (48). Moreover, soil microbes convert arsenic to the most toxic inorganic forms (49). From soil, arsenic seeps into the nearest water tables (50) and, consequently, pollutes the environment.

A significant elevation of arsenic in eggs and excreta resulted from the relative increment of arsenic in drinking-water and/or feed. The strong positive correlation between the arsenic contents in drinking-water and excreta supports this finding. However, arsenic in drinking-water is more detrimental to the living being than arsenic in feed owing to its inorganic nature. With the advancement of age, a concomitant increment of arsenic in eggs was observed. This indicates the aggregated accumulation pattern of arsenic. Positive correlation between arsenic contents in eggs and age further justifies the findings.

### Limitations

Possible limitations of these analyses are that arsenic ingested other than feed and drinking-water were not considered during the analyses of data. Amounts of drinking-water and feed consumed by the hens were ignored. Some of these could affect the arsenic concentrations in layer products. However, the contribution of these limitations to the findings seems to be negligible. The effect of long-term consumption of high levels of arsenic through feed and drinking-water were not considered, which warrants further study.

### Conclusions

The results suggest that arsenic-contaminated drinking-water and feed play a vital role in the elevation of arsenic in eggs as well as excreta of laying hens. High arsenic residue in excreta deserves urgent attention due to environmental concerns and possible recontamination of food-chain in case the excreta are used as fertilizer in conventional or organic farming. Although relatively small amounts of arsenic were obtained from eggs, it must be taken into account due to its detrimental effects on consumers, especially because there are multiple sources of simultaneous arsenic exposures. Although the drinking-water standard for arsenic has been reduced, the standard for arsenic residue in eggs has remained unchanged for decades. Moreover, initiatives to reduce arsenic exposure through drinking-water in Bangladesh are in progress. However, mitigation of the resulting catastrophe deserves urgent attention. Further research considering the limitations of this study would help gain a better picture of arsenic exposure among the people of Bangladesh through eggs and excreta of laying hens.

## ACKNOWLEDGEMENTS

The authors acknowledge the financial contributions from the United States Department of Agriculture (USDA)-Foreign Agricultural Service (FAS), USA; Ministry of Science, Information and Communication Technology (MoSICT), Bangladesh.
